# Invisible labour: A qualitative exploration of the professional identity of interpreters working in UK maternity care settings

**DOI:** 10.1177/13634593251393084

**Published:** 2025-11-27

**Authors:** Tabitha Wishlade, Ginny Mounce, Helen Allan

**Affiliations:** 1 University of Leeds, UK; 2 University of Cambridge, UK; 3 Oxford Brookes University, UK; 4 Middlesex University, UK

**Keywords:** maternity care, post-structuralism/postmodernism, discourse and conversation analysis

## Abstract

The work of professional interpreters is frequently misunderstood and mistrusted, leading to its underuse across healthcare settings. In UK maternity services, this failing contributes to the higher mortality and morbidity of women with limited or no English proficiency. Our study explored interpreters’ professional identities and their contribution to the delivery of care in maternity services. Face-to-face interviews with a purposive sample of professional interpreters working in maternity settings were conducted and data analysed using a version of Foucauldian discourse analysis. Our interpretation of the data is that discourses of ‘women’s work’ were used in constructing the interpreters’ professional identity. Their daily working practice included affective, social and supportive behaviours; however, their subject positions were unrecognised in the voluntary professional codes of conduct for interpreting practice and their labour remained largely invisible and under-valued. Recognising professional interpreters’ identity as invisible labourers suggests that they negotiate biomedical understandings of healthcare interpretation work held by healthcare professionals and women. It allows a more nuanced understanding of interpreters’ practice within maternity settings. Making their work visible offers greater opportunity for regulation, monitoring and evaluation, resulting in greater confidence in its quality and promoting increased uptake.

## Introduction

Over 5 million (9%) people in England and Wales do not have English as a first language, and just over a million of them, approximately 2%, have limited or no English proficiency (ONS, 2022). Limited language skills restrict individuals’ access to public services and they face poorer health outcomes than their English-speaking counterparts (Alexander et al., 2004; NHS, 2015). In view of this inequity and to support efforts in provision of culturally competent care, National Health Service (NHS) providers are obliged to meet the language needs of patients at point of delivery (NHS, 2018). However, while NHS spending on interpreting and translation services is not insignificant, an estimated £174 million between 2019 and 2022 (TaxPayers’ Alliance, 2023), data suggests that this provision is not in place or utilised for all healthcare encounters which require it (MacLellan et al., 2024).

## Background

### Language barriers in maternity

Over a third of births in England and Wales are to non-UK born mothers, a trend that is generally increasing (ONS, 2023), making healthcare interpreting particularly relevant to maternity services. Using professional interpreters to ensure effective communication in the maternity domain not only underpins nursing and midwifery professional codes advocating the recognition of diversity, shared decision-making and informed consent (Nursing & Midwifery Council (NMC, 2018) but is essential for the delivery of safe and ethical care (Cramer, 2017). Several national MBRRACE-UK reports into maternal deaths have shown Black, Asian and minority ethnic women^1^ and their babies to be at disproportionally higher risk of death and morbidity in pregnancy, childbirth and postnatally, in comparison to their White counterparts (see series Knight et al., 2021) and explanations for this include language barriers as contributing factor. Previously, 26 maternal deaths in the UK between 2006 and 2008 were linked to inappropriate interpreting services being provided to women along the pregnancy continuum (Cantwell et al., 2011). This picture remains poor; the recent MBRRACE-UK annual report of maternal mortality and morbidity for 2020–22 shows language support for migrant women to be inadequate throughout their care, and professional interpreter services unavailable at many interactions, both scheduled and emergency (Felker et al., 2024). A recent longitudinal UK study of eight non-English speaking pregnant women described their lack of choice of interpreter and dissatisfaction and distrust of the service they provided (Rayment-Jones et al., 2021).

Numerous UK studies detail the challenges faced by users of maternity services due to language barriers (Ali, 2004; Alshawish et al., 2013; Binder et al., 2012; Cross-Sudworth et al., 2011; Davies and Bath, 2001; Jomeen and Redshaw, 2013; Lam et al., 2012; McCourt and Pearce, 2000; Moxey and Jones, 2016; Phillimore, 2016; Puthussery et al., 2010) and similarly for other non-UK regions (Small et al., 1999, 2014; Watson et al., 2019). Reported harmful effects include under-reporting or misinterpretation of symptoms, missed appointments and women undergoing procedures without full consent (Ali, 2004; Alshawish et al., 2013; Beake et al., 2013; Bridle et al., 2021; Bulman and McCourt, 2002; Cardwell and Wainwright, 2019; Davies and Bath, 2001; McAree et al., 2010). It is known that using appropriate interpreting services can improve the experiences of women’s involvement in maternity care not just in relation to consent but also enabling them to seek reassurance and exchange information (Karliner et al., 2007; Yelland et al., 2017). NICE (2023) clearly states the services of an interpreter be available for pregnant women where needed, and principles for improvements in translation services are set out in NHS England guidance (2018). The expectations for interpreters are governed by their employer; and for the majority of interpreters working in maternity settings in the UK this is the NHS (2018). As a condition of employment interpreters will be required to demonstrate appropriate qualification or training, and may choose to join the UK National Register of Public Service Interpreters (NRPSI). This is the independent ‘voluntary regulator of professional interpreters specialising in public service’. All interpreters registered with NRPSI have met their standards for education, training and practice in public service and ‘all registrants are subject to the NRPSI Code of Professional Conduct’ (NRPSI, 2016). However, it should be clear that the NRPSI codes are voluntary ideas of best practice and not a condition of NHS.

In spite of this, professional interpreters are systematically underused by clinical staff and perceived as inflexible, time-consuming, inaccessible and costly (Baurer et al., 2014; Bischoff and Hudelson, 2010; Jimenez, 2009; Yelland et al., 2016). Clinicians^2^ may misunderstand the depth, and mistrust the quality, of interpreters’ practice. For example, midwives have cited instances where information was not communicated accurately by interpreters (Aquino et al., 2015) although their strategies for assessing this may be based only on their interpretations of non-verbal clues such as tone and body language (Dueweke et al., 2016). Perceptions of interpretation as poor can make clinicians reluctant to use interpreters’ services (Jimenez, 2009) or to believe they can ‘get by’ without interpreters, relying instead on gesturing, their own limited language skills or documented patient histories (Diamond et al., 2009). Yet these perceptions are inaccurate: a professional interpreter’s service not only improves communication and reduces mistakes between language discordant clinicians and patients (Flores, 2005; Karliner et al., 2007; Whitaker et al., 2022) but may also lead to increased patient and clinician satisfaction (Bagchi et al., 2011) and more patient-centred care (Karliner et al., 2011). Professional interpreters are not perfect and can make interpreting errors, although these are less likely to have clinical consequences than when ad-hoc or no interpreter is used (Flores et al., 2012). Professional interpreters potentially reduce costs to health systems in the long term (Bischoff and Denhaerynck, 2010; Jacobs et al., 2004; Lindholm et al., 2012).

If the maternal morbidity and mortality associated with poor provision of language services is to be avoided, clinicians must recognise the contribution professional interpreters make to high-level patient care. One factor moderating this contribution is interpreters’ professional identity, which has a direct bearing on their job performance (Sela-Sheffy and Shlesinger, 2011). This study examines the professional identity of interpreters working in UK maternity settings to highlight their contribution to providing quality care along the pregnancy continuum and promote their use throughout the maternity domain. This includes interpreting for pregnant women in settings such as antenatal clinics, seeing a midwife or when in labour. We will refer to them throughout as maternity interpreters. Like other work that is undervalued aspects of interpreting including the emotional labour, and voluntary administrative and advocacy activities, interpreting can be considered ‘invisible labour’ (Kunreuther and Rao, 2023) and we use this concept when we consider the work done by professional interpreters.

### Professional identity

Professional identity is an individual’s image of themselves as a professional, based on their attributes, beliefs, values, motives and experiences (Ibarra, 1999). Additionally, while partly based on an individual’s role (Gee, 2000), it is formed within a social context (Lawler, 2014), meaning any view the individual has of themselves as a professional relies on their perceptions of others’ image of them as a professional (Jenkins, 2014). In clinical settings social identity theory indicates that professional groups tend to view other groups as irrelevant, or less favourably than their own (Bochatay et al., 2019). Nevertheless, occupational groups with roles deemed ‘professional’ are accorded higher social status (HCPC, 2014). A professional is defined as ‘a person who has the type of job that needs a high level of education and special training’ (Cambridge University Press, n.d.) with a key marker being professional regulation (HCPC, 2014). Accordingly, the NRPSI maintains a register of appropriately qualified and competent interpreters who meet their standards. The NRPSI promotes the professional standards of its members through a code of conduct and will investigate any complaints about their conduct or competence (NRPSI, 2016). The NRPSI Code of Conduct (2016) makes clear that the role of interpreters is to interpret ‘truly and faithfully what is uttered, without adding, omitting or changing anything; in exceptional circumstances a summary may be given’. They are expected to have ‘an adequate level of awareness of relevant cultural and political realities in relation to the country or countries concerned’ although it is not stated how this knowledge could be applied. It is important to emphasise here that there is no single statutory code for interpreters in the UK. NRPSI is a voluntary professional body and membership of NRPSI is not mandatory for employment as a healthcare Language Interpreter by the NHS. However, typically essential NHS role requirements included holding an appropriate qualification for example, Diploma in Public Service Interpreting (Health) and demonstrable understanding of the role of the interpreter, ethics and boundaries which could be by reference to the NRPSI code of conduct for interpreters. Recently, updated requirements have been published with the latest Improvement Framework (NHS England, 2025). Typically, and as the case for our study participants, the role is to provide professional, accurate and effective interpretation on behalf of Health and Social Care professionals, facilitating communication between patients/service users and healthcare professionals who do not share a common language. Furthermore, duties are to provide a true and faithful interpretation between parties and to observe impartially whilst interpreting. The NHS, as the institution using interpreting services, makes clear that interpreters should interpret for everyone present in an appointment, and ‘only to facilitate communication during the appointment. They should not be asked to undertake additional/ ancillary duties during the appointment (e.g. those which may be delivered by a carer or advocate)’ (NHS, 2018: 9). Interpreters do not have the same scope of practice as others such as bilingual health advocates, who are also expected to give patients active and practical support in an advocacy role (El Ansari et al., 2009). A local UK framework for advocacy services states that ‘the role of the interpreter is recognisably different from that of an advocate, as an interpreter is paid to be impartial and communicate a message between professionals and users’ (Silvera and Kapasi, 2002: 37).

Professional identity reveals an individual’s ‘attitude, affect and behaviour’ (Caza and Creary, 2016: 4) which has implications for their standards of practice. Professional identity can therefore be understood as one of the factors that moderates the service professionals provide. While it is linked to a professional’s role it is not synonymous with it: role can be understood as what professionals ought to do, but it may not reflect what and how they actually practice. Therefore, to understand what it is that maternity interpreters do, this study explored the construction of maternity interpreters’ professional identities.

### Literature review

Initially a literature review of healthcare databases was conducted. No literature was found to focus solely on interpreters’ professional identity in maternity settings; therefore, the search results included papers that explored the interpreters’ role across all healthcare fields and global regions. Included papers were judged to be valid after assessment by the Critical Appraisal Skills Programme Checklist (CASP) for qualitative research tool (CASP, 2018).

The review found the global landscape of healthcare interpreting to be complex, encompassing often contradictory expectations, guidelines and practices and affected by the structural constraints of institutions as well as the personal orientation of the interpreter. Commonly, the main role of the healthcare interpreter is traditionally viewed as that of linguistic conduit, passing information between clinicians and patients without inhibiting the therapeutic clinician-patient relationship (Dysart-Gale, 2005; Fatahi et al., 2008; Hsieh, 2006; Rosenberg et al., 2007). This conduit model is quite strongly imagined in the UK (Wang, 2017) and yet interpreters work may go beyond this. For example, although linguistic accuracy is of primary concern for many clinicians (Angelelli, 2004; Dysart-Gale, 2005; Robb and Greenhalgh, 2006; Wiking et al., 2009), interpreters often act as intercultural communicators, to ensure meanings transcend both linguistic and cultural differences (Hale, 2007; Wadensjö, 1998). They may limit miscommunication by clarifying meanings (Baraldi and Gavioli, 2012; Kamara et al., 2018; Mirza et al., 2017), use analogies and language correlating to patients’ educational level (Greenhalgh et al., 2006; Kaufert and Koolage, 1984; Wiking et al., 2013) or intervene to explain and illustrate what clinicians mean or expect (Raymond, 2014).

As informational gatekeepers they can guide and keep encounters ‘on track’ (Davidson, 2000: 400), others may edit patients’ narratives or emotional statements (Baraldi and Gavioli, 2012; Greenhalgh et al., 2006); or advise patients what information to give clinicians (Hsieh, 2007; Kaufert, 1999; Mirza et al., 2017). Sometimes their agency, meaning their own choice to take action, is disregarded and unnoticed by healthcare professionals (Farini, 2013; Hsieh, 2007; White and Laws, 2009) although at other times their additional input is actively sought by clinicians (Leanza et al., 2015; Raval, 2003) and acknowledged by patients (Costa and Briggs, 2014). At times their kindness, respect and empathy, enable trust to be built between clinician and patient (Hadziabdic et al., 2009; Nailon, 2006) and provide emotional support beyond healthcare (Hadziabdic and Hjelm, 2016; Hsieh and Hong, 2010; Rosenberg et al., 2007). Some interpreters become cultural mediators through explaining the significance of patients’ cultural background to clinicians (Butow et al., 2012; Kaufert and Koolage, 1984) or informing patients of expected medical norms (Greenhalgh et al., 2006; Rosenberg et al., 2008)and in parts of Europe, including Spain, France, Germany the understanding of the term interpreter overlaps with that of intercultural mediator (Nikitina and Montenovo, 2023). This complex role involves analysis of their interlocutors’ intentions (Butow et al., 2012; Kaufert and Koolage, 1984). At times the co-construction of meaning undertaken by interpreters may be both highly subjective and also political (Angelelli, 2004).

For Davidson (2000), interpreters’ professional purpose as institutional gatekeeper includes an ability and willingness to help each interlocutor equally, but clinicians can view them as agents of the health system (Hsieh, 2006; Kale and Syed, 2010; Robb and Greenhalgh, 2006;) and interpreters’ actions benefit providers more than patients (Leanza, 2005). However, at times their neutrality can be tested by loyalty to their shared cultural affinity (Kaufert and Koolage, 1984; Mirza et al., 2017) and they may act more as advocates (Baraldi and Gavioli, 2012; Butow et al., 2012; Greenhalgh et al., 2006; Hsieh, 2006; Leanza et al., 2015; Mirza et al., 2017; Wiking et al., 2013). Nevertheless, however strong their professional purpose and affinity with patients, their role as interpreters is often constrained both by a lack of time, by clinicians’ ultimate authority (Greenhalgh et al., 2006) and possibly their own self-regulation arising from their desire to maintain their neutrality or conduit role (Hsieh, 2008).

Overall, the literature shows the role of public service interpreters working in healthcare settings is shaped by several possibly contradictory factors all of which are informed by ideas of power: the power of interpreters to exercise agency and apply their subjectivity in interpreted encounters; the power interpreters wield on behalf of the healthcare institution or patient; the power dynamics between interpreters and clinicians; and the power interpreters exercise over themselves in terms of self-regulation. This idea of power informed our chosen methodology and analysis as we sought to discover how maternity interpreters in the UK constructed their professional identity. The study is reported following SRQR guidelines (O’Brien et al., 2014).

## Methodology

Using a poststructuralist approach this study sought to gain a deeper insight into interpreters’ professional identity construction. Poststructuralists reject the notion of fixed identities, preferring the terms ‘subject positions’ or ‘subjectivities’, although these concepts are arguably equivalent. Foucault (1972) resists the concept of identity as it may essentialise the person (Gibson, 2013), yet identity can be conceptualised as ‘contingent, provisional [. . .and] always in process’ (Townley, 1993: 522). As such, identity and subjectivity are used interchangeably by various authors (see Burr, 2015; Gibson, 2013; Wright, 2004), which the first author adopted in this study.

Poststructuralism holds that the meanings of objects and social practices are not decided by individuals but are constituted by discourse, which are historically and culturally-specific statements that name, describe, explain and judge (Foucault, 1972). Foucault does not posit an ontological distinction between objects and social practices (Wetherell et al., 2001). An object has no innate meaning but language produces all meaning of that object (Weedon, 1987). Discourse is not equivalent to language but as Wright (2004: 36) highlights: ‘[C]hoices in language [. . .] point to those discourses being drawn upon by writers and speakers, and to the ways in which they position themselves and others’. It therefore follows that language (and by extension discourse) constructs identity: ‘It is in the process of using language – whether as thought or speech – that we take up positions as speaking and thinking subjects and the identities that go with them’ (Weedon, 2004: 18). Since discourse constructs identity, an examination (by discourse analysis) of its use in social and cultural contexts can aid understanding of worldviews, relations and identities (Paltridge, 2012). For poststructuralists, meanings remain fluid and context specific and so identities are not singular or unchanging; poststructuralism holds that individuals exist within ‘overlapping, intersecting, often contradictory discourses [. . .] a constant performance of shifting, plural and often discordant combinations’ (Jackson, 2009 in Grant, 2014: 546). Foucauldian discourse analysis moves beyond ascertaining linguistic meaning of interactions; it is concerned with the ways in which power imbalances, both restraining and enabling, comes to shape dominant versions of social or institutional practices (Khan and MacEachen, 2021). This analytic approach makes interpretation contingent, and findings subject to scrutiny (Graham, 2011). In this regard we took care to engage with reflexive practice, including sharing out subjective thoughts and opinions throughout and make no claims to a production of ‘truth’.

## Study design

This study aimed to examine the professional identity of interpreters working in maternity services (maternity interpreters) to highlight their contribution to providing quality care along the pregnancy continuum and promote their use throughout the maternity domain. Sampling was purposive and nine interpreters with experience of working in maternity settings in the UK were recruited as participants by responding to advertisements at participating NHS hospital trusts.^3^ Participant characteristics are shown in Table 1. Two participants (i7 and i9) were employed both by an external agency and an NHS Trust; with the agency work as cover for absent staff at their main employing NHS Trust. The expectations for their role as interpreters were determined by NHS (2018). All the participants were women.

**Table 1.** Participant characteristics.

Participant	Interpreted language	Years of experience	Employer	Number of hospital trusts
1	Romanian	>15	NHS Trust	1
2	Urdu	10–15	NHS Trust	1
3	Farsi/Dari	5–10	NHS Trust	1
4	Farsi/Dari	>15	NHS Trust	3
5	Farsi/Dari	10–15	NHS Trust	3
6	French	<5	NHS Trust	3
7	Urdu	10–15	NHS Trust & external agency	3
8	Portuguese	5–10	NHS Trust	3
9	Polish	10–15	NHS Trust & external agency	3

Semi-structured interviews were held with the participants between April and July 2016, a pragmatic period of data collection although the sample size was as anticipated. Written informed consent was obtained prior to the commencement of the interviews, which were conducted in participants’ homes or places of work and lasted around an hour. The first author was a midwifery student with no prior relationship with participants. Interviews were recorded and the audio data transcribed and anonymised before being analysed according to a modified version of Willig’s approach to Foucauldian Discourse Analysis (Willig, 2012). Discussion of the findings by the first (student) author with experienced qualitative researchers added credibility by rehearsal of reasoning and theory. The resulting findings are presented as identity constructs corresponding to the discourses employed by the interpreters to explain their professional work.

### Ethical considerations

Ethical approval for the study was received from the School of Healthcare Research Ethics Committee (SHREC) at the University of Leeds in March, 2016. Local governance approval was subsequently sought from each of the three participating NHS Trust’s Research and Development departments.

## Findings

The interpreters adopted various subject positions in discourses explaining their role; their identity was therefore constructed as a gendered profession through normalised descriptions of the qualities or functions of ‘women’s work’ (Haines et al., 2016). Our post-structural approach means this interpretation was not necessarily ‘acceptable’ or conscious to the participants; it was their discourses rather than the interpreters themselves we focused on in data analysis. We produced constructs through analysis of discourses as researchers. As researchers we consciously and reflexively worked to interpret their discourses rather than reproduce those power relations familiar to us in maternity and childbirth settings. These identity constructs are presented, together with an explanation as to how they pertain to the meta-discourse of ‘women’s work’ which is discussed later.

### With women/midwives

Frequently, interpreters construct their identity as maternity interpreters with reference to the individual needs of women. A discourse of ‘the vulnerability of pregnancy’ is used to show how women’s emotional, psychological and cultural sensitivity along the pregnancy continuum requires interpreters to give them specific support:

‘I love it when I go and help the [pregnant] women who are usually very emotional. I like to empathise with them and try to understand them’, (i5)

‘Especially in pregnant woman, they are worry like: “Size of my baby is not normal: wha’,s going to happen?” [. . .] So I trying to calm them down, gave my knowledge to them. They really appreciate that.’ (i3)

‘I’,s different with midwives. Some things are offensive to [a] pregnant woman’s culture. And if the midwife is upsetting the patient and she asks I said, “’,m sorry, I don’t think this is the right way of doing it because this is her culture”. If she’s pregnant and fasting, if she doesn’t fast it doesn’t make her feel better, it makes her feel worst.’ (i5)

In responding to women’s individual needs (through offering women advice, reassurance, emotional support and cultural advocacy) the interpreters practise is woman-centred – the discourse underpinning midwifery practice. This familiarity is valued:

‘When I was working with the midwives. I feel like the whole family were grateful that I turned up and they had a familiar face. They knew that [. . .] I wouldn’t lie to them.’ (i1)

‘If there was an emergency, they would call and I would be like, “I’,s ok, phone the hospital”. Because I had that knowledge and I was confident to say, “I’,s ok, you’ve got contractions, wait half an hour then write it down.”’ (i9, NHS Trust & external agency)

Although they can be aware such actions are beyond their scope of practice (of interpreting), interpreters justify this identity construction by highlighting how they perceive maternity services are failing women:

‘And the mums – they do’,t feel as supported, they don’t have the knowledge, they don’t know who to go, who to ask.’ (i9, NHS Trust & external agency)

Being ‘with women’ belongs to a meta-discourse of ‘women’s work’ as typically related to the midwifery profession, and it also entails (or is thought to entail) functions of ‘women’s work’, including supporting and reassuring.

### The routine worker

Other interpreters differentiated working in maternity services from other areas of healthcare, constructing their identity as routine workers within the maternity domain. They develop and display a familiarity of interpreting within maternity services; by following repeated events such as antenatal consultations whenever they work with pregnant women, they know the field inside out:

‘[I]’,s all over and over again. Nothing different [. . .] With a pregnant woman, it’s the same thing.’ (i4)

‘’,ve learned the whole process of how everything works [. . .] And now like I think the booking appointment is on my tongue tip. I know the questions.’ (i8)

This is not merely acquaintance with processes, it is routine (in the sense of ordinary and typical) work, which by custom is ‘women’s work’.

This proficiency opens the possibility for them to be entrusted with undertaking actions that are normally the preserve of midwives (themselves doing ‘women’s work’) such as explaining postnatal information leaflets to women:

‘Because the staff knew I knew it so well, they would give me the leaflets and ask me to [explain] them [to the women]’, (i1)

While these actions are outside the scope of their role as the NHS describes, but enables them to highlight to midwives what they have missed when providing care, which is seen as a way to protect women:

‘Sometimes they [midwives] go fast but the women does’,t know what they are saying. They just want to leave. It’s risky for the woman. So I say, “What about this”, (i2)

The identity construct of the routine worker belongs to a meta-discourse of ‘women’s work’ because it draws on normalised ideas about the qualities of ‘women’s work’ (being routine) and encompasses a function of such work (protecting).

### Community helper

The interpreters deploy a discourse of ‘giving back’ to construct their identity as socially-engaged professionals serving their community: *‘’,m being active, I’m contributing something to my community’* (i5). The implication of this subject position is that interpreters volunteer to do tasks for women that are not in their job description (unlike that of a bilingual advocate as described earlier in *professional identity*) because of their shared community membership: ‘*We do these things [extra tasks] to serve the community people’* (i2). This includes helping women with navigating the hospital; making phone calls and reading letters for them; providing women with emotional support by listening to their fears and giving them counsel; and doing private interpreting for them without charge:

‘The patients usually say, “If we need you other places, you come and we will pay you”, And my answer is, “You call me; if I am free, as a friend, I come”,’ (i5)

The interpreters are providing voluntary assistance, but what they describe are aspects of work which are routinely and historically performed by women.

The identity construct of the community helper belongs to a discourse of ‘women’s work’ because it involves the functions of helping, supporting and counselling, but also because it displays one of its qualities, which is that it is unpaid. These are all normalised attributes of ‘women’s work’ (e.g. DeVault, 1991).

### The appeaser

Interpreters use a discourse of accountability to construct their identity as appeasers. In this subject position interpreters are answerable to either the institution or themselves for what happens to women and therefore encourage patients to receive an adequate standard of care. For example, when a woman does not know why she is going into theatre, an interpreter insists the consultant comes to explain the procedure because she feels she may be blamed by the institution for the woman’s ‘ignorance’:

‘So they brought the consultant in who was annoyed. Because it could come back to me; my name goes on the form.’ (i6)

Interpreters also use a discourse of accountability to demonstrate how patients hold them responsible for healthcare processes: *‘Sometimes they would get angry with me because I was the one saying i’*, (i1). Because interpreters are intelligible to patients, they – and not the clinicians for whom they interpret – are seen to be creating the patient’s reality. Therefore, interpreters adopt the subject position of appeaser in a second way – to mitigate any offence caused to women by the healthcare system. In this position they either modulate the words or tone of an interpreted message so that the patient *‘can understand and still you know she wo’,t feel offended’* (i2) or apologise and reassure patients about long waiting times because ‘*there’s nothing worse than a patient who comes angry at you to the appointment’* (i9, NHS Trust & external agency dual). Interpreters’ view adopting this version of the appeaser as enhancing communication with, and the co-operation of, patients in the interpreted encounter.

The subject position of the *Appeaser* belongs to a meta-discourse of ’women’s work’ as it engenders functions oriented to this discourse (appeasing and facilitating).

### The patient and counsellor

The emotional work of appeasing is burden which is reflected in the next construction of the interpreter’s identity, that of being vulnerable to illness. A discourse of pathogenesis is used by interpreters when discussing their susceptibility to, and recovery from, the trauma they experience through their profession. Interpreters are left run-down and thus susceptible to illness owing to the taxing and emotional nature of their job: ‘*It’s emotional and exhausting. Interpreting takes all the energy*’ (i3). They are then overwhelmed by the emotions they encounter at work, leading to a diseased state: ‘*All these different energies, they will get to you. I did become ill. Emotionally*’. (i4). But interpreters can learn, over time, to guard against becoming ill – ‘*I have to learn to protect myself*’ (i3) – by reducing their hours or refusing work they find too emotional. They also deploy a discourse of the talking cure to show how they treat such attacks:

*‘*I do find I get sometimes emotionally down but then I come to the office and I talk to my colleagues [. . .] and then the’,ll calm me down.’ (i8)

Through the discursive performance of the talking cure, their identity is constructed as both patient and counsellor to one another and here this identity construct of the *Patient* belongs to a meta-discourse of ‘women’s work’ because it is a result of one of the qualities of such work; that it is mentally and emotionally demanding. The identity construct of the Counsellor belongs to a meta-discourse of ‘women’s work’ in which normalised functions of ‘women’s work’ – caring and listening – are carried out.

### The tool of empowerment

As indicated, our participants were all women, and at least some see themselves as political agents whose professional motivations centre around a discourse of female empowerment:

‘Absolutely interpreting is a political act! I have a very strong opinion that women has to be independent.’ (i5)

Although this orientation can lead to overtly political actions (again, beyond their role) such as giving women the contact details of refuges, encouraging them to speak English or discussing removal of the hijab, just the presence of the interpreter is seen as a feminist discursive practice as she displaces a man as the woman’s voice:

‘Without interpreters, sometimes the [male] family members you know they force them [women] to say things’ (i2).

Enacting feminist discourse in this way (e.g. calling out/recognising social practices that reinforce gendered power) constructs the interpreter’s identity as a tool of female empowerment, although power differences in the encounter are not only gendered (Hsieh, 2013). In this subject position, the interpreter informs women of their right to appropriate care within the medical system:

‘[T]hey [the women] won’t complain [about poor care] because they don’t have confidence and they need the midwife, but I know the usual process and I tell them.’ (i9, NHS Trust & external agency)

Furthermore, interpreters nurture women throughout their repeated interactions, helping them develop confidence:

‘They are very shy [. . ..] I always tell them they do well. That baby is well. Then gradually, you see them start to feel more confident and maybe speak a little English.’ (i7, NHS Trust & external agency)

The subject position of the tool of empowerment belongs to a meta-discourse of ‘women’s work’ because it contains a function of such work (nurturing) which once again is normalised gendered role (Eagly and Crowley, 1986) for women professionals.

### The sanctioned transgressor

In our findings many of the subject positions entail the interpreter acting in ways that are outside her scope of practice (described earlier in *Professional identity*) defined by her employer the NHS (2018). Neither are they supported by the voluntary code of practice NRPSI (2016). However, interpreters use a discourse of ‘turning the blind eye’ to construct their identity as sanctioned transgressors. In this subject position it is the clinicians who allow interpreters to do extra tasks so long as they help to meet their own objectives:

‘The lady was quite unfriendly [. . .] and they just not getting anywhere. And she was just swearing and cursing at them all. Any interpreter she had I think everything she said it was translated. So when I started working [. . .] I just did’,t interpret any of the words. I would just water it down, say something nice and polite. And they [clinicians] knew what I was doing. But because the way I interpreted [was polite] so the way they answered was nice, better. So gradually it improved.’ (i4)

In this example clinicians are aware the interpreter is modifying what the woman says but allow her to do so because it makes communicating with her easier. In other versions of the sanctioned transgressor, clinicians do not just accept these transgressive behaviours but actively promote them. This is also seen in the identity construct of the routine worker, wherein midwives ask interpreters to explain information leaflets to women by themselves. They also expect interpreters to guide patients around the hospital; help them to navigate trips to the pharmacy; and keep patients calm so they can complete the appointment. These are all actions which exceed their official role requirements. Thus, clinicians allow or expect interpreters to become sanctioned transgressors not only for their benefit but also for that of the patient:

‘They [clinicians] want you to do everything to make it [providing care] easy for them and better for the patients’ (i6).

This identity construction gives interpreters a feeling of security about the tasks they undertake for women: ‘*If they [clinicians] don’t mind then why should I mind*?’ (i4). Although this subject position does not necessarily open avenues of action for the interpreters, the security it gives means they try to gain sanction from clinicians for the extra responsibilities they take on:

‘So then I seek the professiona’,s permission, and if we have to do anything to help her then we do it there and then in the clinic. So that’s very professionally done.’ (i8)

The sanctioned transgressor belongs to a meta-discourse of normalised ‘women’s work’ as it contains the functions of facilitating, guiding, communicating which are to do with communality; characteristically the female stereotype (Conway and Vartanian, 2000) Moreover, ‘women’s work’ is often not officially recognised for the work that it is, despite being necessary to keep systems functioning effectively. As sanctioned transgressor, interpreters act in ways that allow patients to receive good care and clinicians to fulfil their objectives (even though this work does not form part of their code of practice) and this identity construct also demonstrates a quality of ‘women’s work’.

## Discussion

This study aimed to explore how the professional identity of maternity interpreters in the UK was constructed through our analysis of their discourses. The findings show that participants adopt subject positions that exist within a discourse of ‘women’s work’, and that they do this in three ways. Firstly, they construct their identity in terms of other (typically) gendered professions such as midwife or counsellor. Secondly, they construct their identity in accordance with perceived qualities of ‘women’s work’ such as being routine (Gregory and Windebank, 2000); being done voluntarily (Hakim, 2016); and being mentally and emotionally demanding (Hochschild, 2012). Finally, the interpreters construct their identity in terms of the normative functions of ‘women’s work’. These centre on socialisation, caring and affective behaviour (Goodman, 2013), but also include ‘nurturing, mediating, organizing, facilitating [and] supporting’ (Acker, 1989: 219). These functions are most obviously present when interpreters give emotional support and advice to women; give one another counsel; facilitate communication by acting as appeasers; and nurture women.

Abbott and Meerabeau (1998) among others (Daniels, 1987) argue that ‘women’s work’ is regarded as an expression of women’s natural roles and as such the skill it takes is consistently unrecognised or unappreciated. This is reflected in both their formal role and voluntary interpreters’ codes of conduct, which do not recognise this aspect of interpreters’ practice (see NRPSI, 2016). Accordingly, by deploying ‘women’s work’ as a meta-discourse the interpreters construct an overarching identity of themselves as invisible labourers. This is not to suggest their work is always underappreciated: as the identity construct of the sanctioned transgressor highlights, clinicians are – at least part of the time – aware of this work and occasionally actively encourage it, either for their own or the woman’s benefit. What this overarching subject position does highlight is that current interpreting standards (specifically job roles) do not correlate with maternity professional interpreters’ practice; while some of their actions may fulfil a current need in maternity, it may also exceed the scope of the role as set out by employers.

### ‘Women’s work’

This is the first study using discourse analysis to conceptualise some UK public service interpreters’ practice, specifically in maternity settings, as ‘women’s work’. This may be that with an all-female cast of participants talking about their experiences of interpreting for women in a woman-centred domain, the scene is set for a female focused discussion of identity. It may also be because maternity interpreters work within a particular setting which is different to the other areas of healthcare in which previous research into this topic has been based. The biomedical paradigm which pursues objectivity (Solli and Barbosa da Silva, 2018), is most powerful in healthcare (Ashcroft and Van Katwyk, 2016), but in maternity it competes with the paradigm of midwifery-led care (International Confederation of Midwives (ICM, 2017). This approach views childbirth practices as a natural life event, and promotes a woman-centred ideology embracing psycho-social care and is often contrasted with obstetric practice and biomedical ideals (van Teijlingen, 2005).

Our findings demonstrate how maternity interpreters practise ‘women’s work’ by supporting, advising, appeasing, nurturing and advocating. These practices depend upon both interpreters’ and patients’ perspectives, feelings and beliefs and can therefore be considered an expression of subjectivity (Solomon, 2005). Thus, interpreters with experience of working in maternity, where subjectivity (as part of midwifery-led care) is encouraged, may feel meaning male capital in this setting may be lessened, perhaps contributing to their low presence. Similarly, while UK male midwives have an established role, albeit in small numbers, their presence in maternity spaces remains somewhat contested (Pendleton, 2024).

We suggest the identified functions of ‘women’s work’ in the UK context of maternity interpreters make a significant contribution to existing healthcare interpreting literature. Interpreters provide care, comfort and counsel to both patients (Messias et al., 2009; Phanwichatkul et al., 2018) and clinicians (Miller et al., 2005) They listen to, sympathise with, and display empathy to patients (Hsieh, 2006; Mirza et al., 2017; Rosenberg et al., 2007), becoming the sensitive and humane face of clinical care (Leanza et al., 2015). Furthermore, interpreters present themselves as lovely, gentle and caring to build relationships with patients (Greenhalgh et al., 2006; Robb and Greenhalgh, 2006). These relationships allow interpreters to become patients’ locus of support (Miller et al., 2005) and friend (Rosenberg et al., 2007). Interpreters nurture patients by encouraging their self-expression (Farini, 2013) or empower patients by welcoming them into the consultation room so they feel more confident (Leanza, 2005). Interpreters also engage in ‘women’s work’ to facilitate a smooth interpreted event: they work to create a good atmosphere (Wiking et al., 2013); organise the information given to clinicians (Hsieh and Kramer, 2012); ensure no offence or embarrassment is caused to patients (Hsieh, 2006; Williams et al., 2018); soothe patients’ anxiety (Robb and Greenhalgh, 2006); and appease their dissatisfaction (Wiking et al., 2013).

Previous accounts of interpreters do not mention ‘women’s work’, suggesting it may not be universally practised (Dysart-Gale, 2005). However, it may be that ‘women’s work’ *is* practised but that the focus of these other studies renders it invisible. Robb and Greenhalgh (2006) find that when talking in the abstract, interpreters present a professional self-image that correlates with their codes of practice, but when recounting specific instances of interpreting, they reveal actions discordant with this. The studies in which no ‘women’s work’ is relayed focus on either the interpreter’s role or healthcare interpreting models. Role is an abstract set of expectations about the behaviour of those associated with a social position (Scott, 2015). Likewise, models are systems of abstract concepts (Scott and Marshall, 2009). Conceivably, therefore, these studies’ participants focused more on the abstractions of their work rather than their everyday working practices when responding to the researchers, meaning any instances of ‘women’s work’ they perform may not have been revealed.

### ‘Women’s work’ and enhanced care

We found that interpreters adopt subject positions aligned with ‘women’s work’ because they wanted to provide women with the type of care they perceived the women deserve but do not always receive. This correlates with Hsieh and Kramer’s (2012: 158) finding that healthcare interpreters working in healthcare settings systematically intervene in encounters to achieve ‘effective, ethical and culturally-sensitive care’. Although important for all patients, this is particularly pertinent for patients with limited or no English proficiency who receive poorer healthcare than their language concordant counterparts (Ngo-Metzger et al., 2007), but again demonstrates the work as exceeding the boundaries of their prescribed role.

Our findings that the practice of ‘women’s work’ by UK maternity interpreters enhances care in a variety of ways, echoes literature about other healthcare interpreter’s work. The relationships interpreters build with patients may go some way to assist in women feeling more confident to attend medical appointments (Miller et al., 2005). Furthermore, these relationships lead to enhanced diagnoses and treatment: they allow interpreters to gain patients’ trust so that they will disclose more information to clinicians (Hadziabdic et al., 2009; Leanza et al., 2015; Rosenberg et al., 2008). They also make patients more liable to accept clinicians’ recommendations and glean valuable information about patients that can help in the appointment (Hsieh and Kramer, 2012). Additionally, the interpreter’s emotional support is seen as an extension of the providers’ care (Hsieh and Hong, 2010), and without it, clinicians believe their ability to convey their concern for patients is threatened (Nailon, 2006). Furthermore, by suggesting ways to patients in which to express themselves, patients believe interpreters help them be better understood by clinicians (Costa and Briggs, 2014).

### Invisible labour

Work undertaken in response to an employer’s implicit or explicit requirements, which is not recognised as work, is invisible labour (Poster et al., 2016). For example, the necessary supportive work undertaken in some professions (such as nursing), is often neglected or unrecognised (Allen, 2015), allowing other professionals (in this case, doctors) to claim all work outputs (such as a recovered patient) as their own (DeVault, 2014). Arguably, the ‘women’s work’ interpreters undertake is invisible labour because it allows the NHS to deliver care according to its aims (which include promoting equality, and delivering care focused on patient experience; Department of Health & Social Care, 2023), without formal recognition of interpreter’s contribution to it.

No work is innately or universally invisible (Allen, 2015); it is a perspective based on the cultural construction of labour (Budd, 2016). Healthcare can be considered a culture in which, as discussed, biomedicine is the predominant paradigm, where practices extract ‘the person-hood from the illness’ (Michalec and Hafferty, 2015: 193), seeing all bodies as the same and thus believing them all to benefit from the same clinical processes. It believes bodies are not a result of interactions with their social and political environments (Lock and Nguyen, 2010), thereby denying a link between inequity and illness (Ashcroft and Van Katwyk, 2016). Further, biomedicine understands clinicians’ subjectivity to distort their ‘apprehension of absolute truth’ and therefore renounces their opinions, emotions and intuitions (Ashcroft and Van Katwyk, 2016 p.144). Accordingly, biomedical labour is constructed as objective, routine, unemotional and indifferent to social justice. Quality is equated with accuracy. It is perhaps unsurprising therefore that the macro-construction of interpreting labour drafted either under NHS (2015) or with reference to NRPSI (2016) this paradigm renders the more subjective elements of interpreting work invisible.

But what does biomedicine gain from this construction of labour? Some might argue that – with its discourses of progress, truth and neutrality – biomedicine is benign, making it harmless for this paradigm to dictate the terms of interpreting labour. Yet for Foucault, biomedicine is not neutral. Instead, it represents ‘particular perspectives, conventions, and motivations’ (Pylypa, 1998: 23), one of which is to (re-)create conditions in which it can maintain power (Finkelstein, 1990). Although interpreters may replicate this power (see the subject position of the Routine Worker), they can also challenge it; in the subject position of ‘with women’ an interpreter defends a woman’s right to fast during her pregnancy (going against biomedical advice) because eating will affect her emotionally (presumably because of the religious and cultural significance to this woman of undertaking the fast). Therefore, to expose and thus legitimate subjective elements of the interpreter’s labour (such as advocacy) would be – potentially – to invite challenges to its power regime. As such, the biomedical construction of labour is neither neutral nor benign but a tool used to maintain its power.

### Invisible labour, enhanced care and the clinician’s objectives

At a micro-level, clinicians – at least ostensibly – tend to construct healthcare interpreting labour in a reductive, and what may be understood as a biomedical way (Robb and Greenhalgh, 2006) expecting interpreters to be impartial and invisible linguistic agents (Brisset et al., 2014; Sleptsova et al., 2014), echoing the NHS principles for the role (2018). As highlighted by the findings, however, clinicians at times expect interpreters to provide support, invisibilised by employment and compensation processes, to enhance patient care. This includes undertaking ‘women’s work’ in terms of providing patients with comfort, counselling and empathy (Brisset et al., 2014; Messias et al., 2009), but also extends to other forms of invisible labour. For example, interpreters can be expected to provide information to both patients and clinicians (Hsieh and Kramer, 2012; Rudvin, 2007) and give patients practical support (Messias et al., 2009). Furthermore, some interpreters asked to ‘give [clinicians their] opinions and actively intrude in the session to explain potential cultural misunderstandings’ (Rudvin, 2007: 10). Yet invisible labour is also expected to fulfil the clinician’s own objectives as they anticipate interpreters to convince patients to undergo certain interventions (Hsieh and Kramer, 2012). In either case, when this invisible labour is not forthcoming there is evidence clinicians become angry or frustrated (Brisset et al., 2014; Hadziabdic and Hjelm, 2016).

## Conclusions

The understanding as ‘women’s work’ makes a significant contribution to the healthcare interpreting literature but is just one form of invisible labour that interpreters perform. Because it is invisible (their roles and NRPSI Code of Conduct fails to address the advocacy work that public service interpreters working in healthcare settings do) they are usually neither trained nor supported to carry out these forms of labour (Davidson, 2000; Dysart-Gale, 2005). That this labour is not actively monitored or evaluated raises concerns regarding its quality.

Institutions and interlocutors require a guarantee of professionalism from interpreters, which is outlined by their codes of practice, but these codes give such an unrealistic expectation of interpreters’ labour that they may be viewed as a ‘zero-risk fantasy’ (Leanza, 2008: 217), particularly as – for members of NRPSI – they are voluntary. The codes’ lack of meaning and prescription is also highlighted by Hsieh (2006) and as such they do not necessarily improve care, facilitate communication or promote patient empowerment. Indeed, Davidson (2000: 401) sees them as actively dangerous, because of the lack of accountability for the labour performed in interpreted encounters. Therefore, to truly protect all parties, the health system needs to formally recognise the labour entailed in providing quality healthcare to Limited or No English Proficiency patients.

As we have indicated, this will entail re-conceiving interpreting work in ways meaningful to maternity interpreters’ practice, which will necessarily subvert the biomedical framing of the work interpreters do. Recognising ‘women’s work’ as inherent in this professional identity aligns with discourses of midwifery which value relational, subjective and affective care. However necessary, ‘women’s work’ interventions are not unproblematic, for example regarding exceeding professional boundaries (Leanza et al., 2015). The next steps will be to develop the existing standards for this labour, such as those principles of high-quality interpreting set out by the NHS (2018), along with processes for monitoring, training and supporting interpreters in its provision, for example using current mechanisms of employer governance. This resonates with previous calls for new models of interpreting (which may encompass many roles), with concomitant guidance on how to practice these roles ethically (see Dysart-Gale, 2007; Sleptsova et al., 2014).

This study found that maternity interpreters adopt subject positions aligned with ‘women’s work’ either through identity category (e.g. with midwife); component (e.g. routine) or function (e.g. affective, social or supportive work). Adopting these subject positions was seen as necessary in order to provide women with high-quality maternity care and enable clinicians to meet their objectives. However, as this work is not recognised by interpreters’ codes of conduct, it can be conceived of as invisible labour. We discussed how this invisibility may be partly due to interpreting labour being defined within or with reference to the biomedical paradigm.

Public service interpreters working in healthcare settings are systematically underused, which contributes significantly to inequity in maternity care and has devastating consequences for woman and babies. At an individual level, this can be because clinicians do not recognise the depth, nor trust the quality, of interpreters’ practice. By way of exploring maternity interpreters’ professional identity, this study addressed both issues. The depth of interpreters’ work is seen in the ‘women’s work’ – alongside other invisible labour – of affective, social and supportive behaviours that interpreters practise. This means that even if clinicians manage to ‘get by’ without them (in communicative terms), service users do not receive the same level of care they would had an interpreter been present. However, the invisible labour interpreters practise is unmonitored; they are not trained to carry it out and because it is ignored by guidelines, there is no instruction on how to practise it ethically. It is clear that some of the ‘women’s work’ described is outside of the employers mandated role expectations. Our findings are not critiquing interpreters’ practice, but rather suggest that the standards, education and training of the professional body (irrespective of whether individual interpreters are members) do not encompass the realities and social aspects of their practice or compensate for interpreters’ work. We recommend that maternity interpreting work be redefined according to a midwifery-led care paradigm, allowing the ‘women’s work’ maternity interpreters undertake to become visible, letting it be monitored and conducted ethically. This will allow clinicians to both realise the depth of the work interpreters undertake and be reassured of its quality, potentially leading to the greater use of professional interpreters within the maternity domain.

### Strengths and limitations

The study’s strength is its particular focus on maternity settings; the importance of effective communication with women and families throughout the childbearing continuum is a topic of huge public and professional concern, especially in light of the known disparities in care as a direct consequence. The main limitations are due to fact that the empirical data was collected several years ago, and this introduces challenges in analysis, but a recent review the literature suggests that issues with the role of professional interpreters have not substantially altered in the intervening years. What we did not investigate was the specific job descriptions of the participants, meaning that we were concerned with their personal perceptions for their roles and professionalism rather than their adherence to interpreting requirements. It appears that the practice demands for interpreters are sometimes outside of the contractual demands, but these boundaries unclear and the work often unregulated. Further systematic inquiry could usefully focus on this gap that is, detailing what employers truly want and expect from interpreters, including having a fully regulated workforce of interpreters. Future study could focus on how interpreters navigate their roles according to their different employers and if these expectations differ. Documenting the various activities done by interpreters in order to identify exactly which of these are exceeding (possibly detrimentally) the role would be an important step in providing transparency for future investigation and for the profession of interpreters working in healthcare settings such as maternity.

## Notes

1. The published MBRRACE reports used this generally British term to refer to people of colour and so we have reproduced this terminology here, although we acknowledge that all acronyms such as BAME are hierarchical, unrepresentative and reductive.

2. Here meaning medical doctors, midwives and nurses.

3. National Health Service managed hospital providing healthcare services to the public.
